# Segmental Arthroscopic Hip Labral Reconstruction Using a Peroneus Longus Allograft

**DOI:** 10.1002/atn2.70375

**Published:** 2026-10-28

**Authors:** Michael T. Kody, Michael B. Banffy

**Affiliations:** ^1^ Cedars‐Sinai Orthopaedics Los Angeles California U.S.A.

## Abstract

Arthroscopic hip labral reconstruction has become an important tool for treating irreparable labral pathology, particularly in the revision setting. Restoration of the acetabular labrum is critical for hip stability, load distribution, and maintenance of the joint suction seal. Segmental labral reconstruction allows focal defects to be addressed while preserving healthy native labral tissue. This technical note describes a reproducible technique for segmental labral reconstruction using a peroneus longus allograft. The use of a peroneus longus allograft provides consistent graft sizing and avoids donor‐site morbidity. This technique emphasizes a stepwise approach, including appropriate patient selection, portal placement, graft preparation, controlled graft delivery, and secure fixation to restore labral function and stability. Segmental reconstruction is particularly useful for focal irreparable defects and may help restore joint mechanics and the suction seal while avoiding unnecessary circumferential reconstruction.

## Graphical abstract

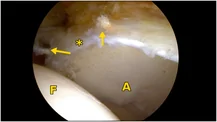

**VIDEO 1:** Arthroscopic segmental hip labral reconstruction using peroneus longus allograft. This procedure was performed on a left hip in the supine position and visualized with a 70° arthroscope. This video shows the surgical technique, including labral debridement, preparation of the anterior and posterior native labral junctions, placement of the anterior reduction anchor followed by a second fixation anchor on the acetabular rim, preparation of the peroneus longus allograft with a ripstop suture at the leading end, graft passage through the posterolateral cannula and reduction to the native footprint, graft fixation, and arthroscopic confirmation of the suction seal. Femoroplasty and capsulotomy closure are performed as indicated. Video content can be viewed at https://doi.org/10.1002/atn2.70375.

The use of arthroscopic hip labral reconstruction has expanded to address numerous hip joint pathologies resulting in an irreparable or deficient labrum.^1^ Restoration of the acetabular labrum is integral to normal hip biomechanics, contributing to joint stability, load distribution, and maintenance of the fluid suction seal.^2^, ^3^, ^4^ Because labral tears are the most common pathology treated in hip arthroscopy, the proper management of labral pathology results in improved patient outcomes.^1^, ^4^ Although suture‐based repair remains the gold standard for most labral tears, irreparable or severe labral deficiency necessitates reconstruction. Depending on the overall state of the acetabular labrum, either segmental or circumferential reconstruction techniques may be employed because both have shown positive short‐ and long‐term outcomes.^1^, ^5^ Although autograft and allograft options can be used, allografts, such as peroneus longus allograft, offer consistent graft size, avoidance of donor‐site morbidity, and technical consistency.^3^, ^6^ Recent systematic reviews indicate that both autograft and allograft reconstructions yield significant improvements in patient‐reported outcomes.^6^

This technical note presents the use of a peroneus longus allograft for segmental labral reconstruction of the hip in a revision setting. Segmental reconstruction is a powerful tool, particularly in revision cases, where focal labral insufficiency can be addressed while preserving healthy labral tissue. This technique presents a reproducible stepwise approach emphasizing effective graft preparation, delivery, and incorporation in a way that can efficiently restore labral function.

## SURGICAL TECHNIQUE

### Preoperative Planning

Preoperative preparation includes a thorough history, physical examination, and imaging studies. Mechanical symptoms, functional limitations, and the onset and nature of pain after hip arthroscopy help elucidate the cause of the patient's pathology. Persistent or recurrent pain after index surgery may suggest technical or structural risk factors for failure. Risk factors include inadequate femoroplasty, capsular deficiency, labral hypoplasia, suboptimal labral repair, or dysplasia.

Clinical examination should focus on hip range of motion, hip strength testing, and provocative maneuvers including flexion, adduction, and internal rotation; flexion, abduction, and external rotation; and log roll maneuver.^1^, ^2^, ^3^ Plain radiographs are important to evaluate and include anteroposterior pelvis view, false profile view, and the modified Dunn's lateral view. In the revision setting, the senior author routinely obtains a magnetic resonance arthrogram to better delineate chondral and labral pathology.

### Patient Positioning

The senior author uses a postless traction table with the patient in a supine position in 0° to 5° of Trendelenburg dependent on body habitus. The patient then undergoes general anesthesia after having received a regional nerve block. The feet are then secured to the traction table in slight internal rotation to match the patient's natural femoral version parallel to the floor. Gross traction is applied to provide adequate hip distraction. Under fluoroscopic guidance, an 18‐gauge spinal needle is used to create an air arthrogram. Then, fine traction is applied for further distraction. The operative extremity is prepped and draped in the typical sterile fashion.

### Portal Placement and Access

First, the anterolateral (AL) portal is created with a spinal needle under fluoroscopic guidance (Table 1, Video 1). A guidewire is placed through the spinal needle into the joint and used for cannula placement. The 70° arthroscope is inserted. With fluid flow off and under direct visualization, the midanterior portal (MAP) is established with a spinal needle, nitinol wire, and cannula. Once both portals are established, flow is turned on, interportal capsulotomy is performed, and diagnostic arthroscopy is completed.

**TABLE 1:** Portal Positioning

**Portal**	**Abbreviation**	**Anatomic Position**
Anterolateral portal	AL	Located 2 cm anterior and 1 cm distal to the proximal tip of the greater trochanter
Midanterior portal	MAP	Positioned approximately 45° distal and medial to the AL portal; slightly medial to the midpoint between the AL portal and a vertical line extending from the anterior superior iliac spine to the midpoint of the knee
Distal anterolateral accessory portal	DALA	Placed approximately 4 cm distal and slightly anterior to the AL portal, creating an equilateral triangle with the AL portal and MAP
Posterolateral portal	PL	Established 4 cm directly posterior to the AL portal

### Intra‐articular Evaluation

The labrum and chondral surface are assessed for pathology (Figure 1). Significant chondromalacia or chondral‐labral pathology may preclude labral reconstruction. Additionally, the anterior and posterior native labral junction sites should be thoroughly assessed to ensure the presence of a healthy labrum on both sides of the segmental reconstruction. In the setting of irreparable labral pathology, a segmental reconstruction is most appropriate for focal defects, involving the anterior to posterior labrum (approximately the 3‐ to 9‐o'clock positions). Adequate healthy labrum must be present at both the anterior and posterior margins to allow secure graft docking. More extensive defects may be better addressed with circumferential reconstruction. In the revision setting, the previous anchor points should be assessed for planning new anchor placement.

**FIGURE 1:**
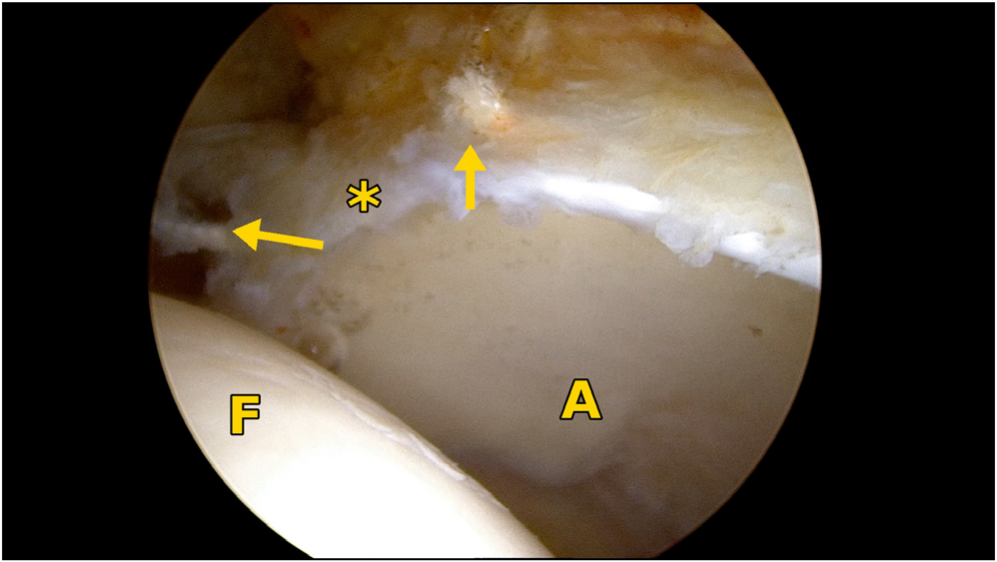
Left hip, supine position, arthroscopic view from the AL portal using a 70° arthroscope. In this revision case, the native acetabular labrum is diminutive and irreparable (asterisk). Two previously placed suture anchors (arrows) are seen along the acetabular rim. The F is seen in the lower left and the A centrally. (A, acetabulum; AL, anterolateral; F, femoral head.)

### Labral Debridement

Once the decision is made to proceed with labral reconstruction, the distal AL portal and the posterolateral (PL) portal are created (Table 1). The distal AL portal is made approximately 4 cm distal and slightly anterior to the AL portal. This is done under direct visualization in a similar manner to the MAP. The PL portal is approximately 4 cm posterior to the AL portal. The location is found with a spinal needle using fluoroscopy and direct visualization. Once all portals are established, the labrum is reassessed. The labrum is debrided using a combination of an arthroscopic shaver, biter, and electrocautery (Figure 2A and B). The acetabular rim is prepared for anchor placement using a 5.5‐mm burr. In the revision setting, previous anchors are removed if they will interfere with future anchor placement. After segmental removal of the diseased labrum, the anterior and posterior portions of the healthy labrum are carefully prepared for segmental reconstruction. At this time, electrocautery can be used to shape the ends of the labrum (Figure 3). Each end should be resected to healthy, viable tissue with normal morphology suitable for graft integration.

**FIGURE 2:**
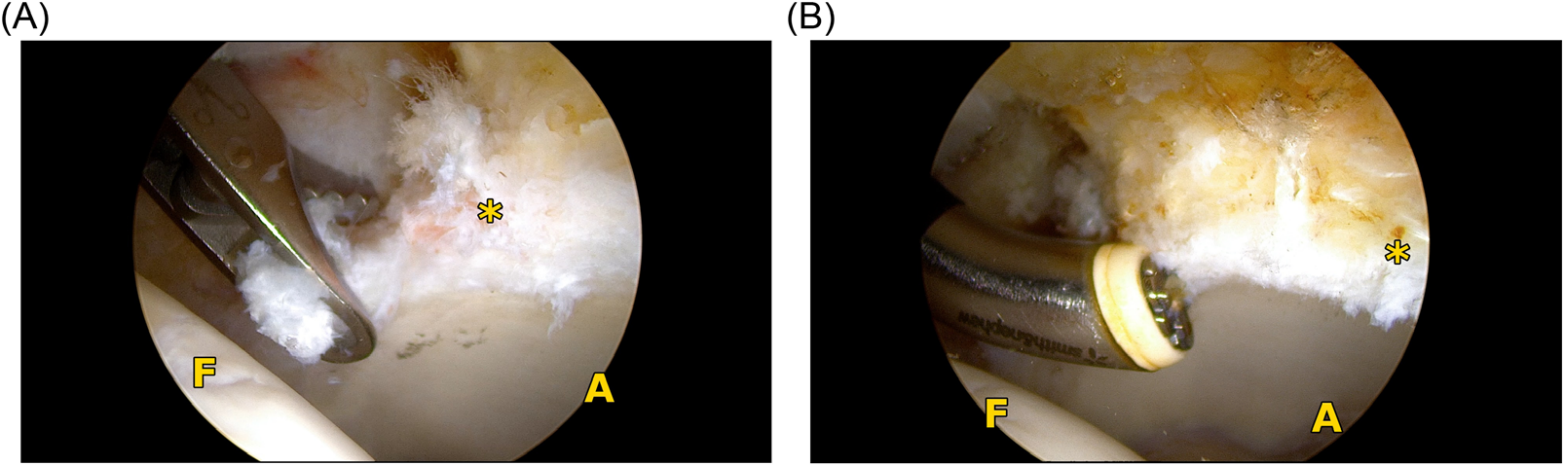
Left hip, supine position, arthroscopic views from the AL portal using a 70° arthroscope. The degenerate, irreparable labrum (asterisk) is debrided to the acetabular rim. (A) An arthroscopic biter is used to resect the degenerate labral tissue. (B) Electrocautery is used to prepare the acetabular rim for anchor placement. The F is seen at the lower left and the A at the lower right. (A, acetabulum; AL, anterolateral; F, femoral head.)

**FIGURE 3:**
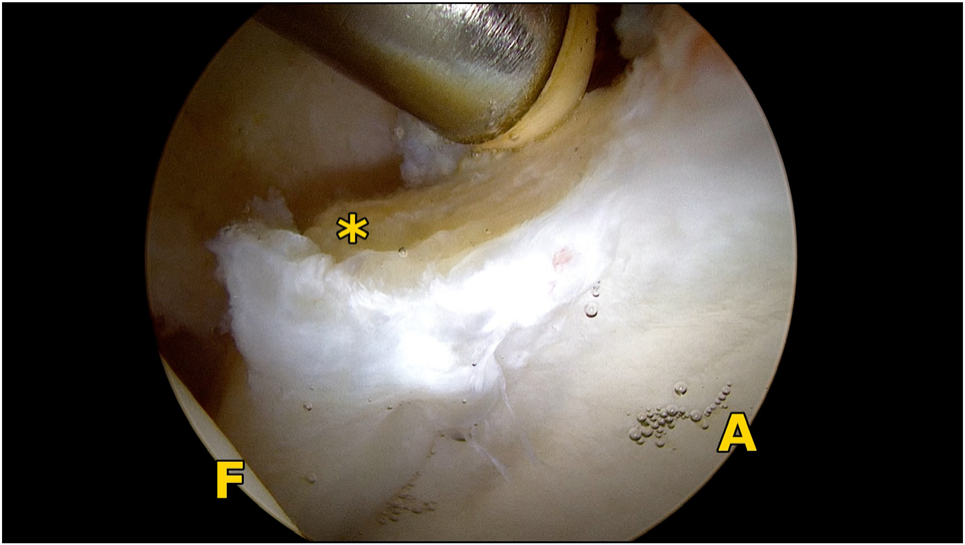
Left hip, supine position, arthroscopic view from the AL portal using a 70° arthroscope. The anterior junction of healthy, viable native labrum (asterisk) is prepared and shaped with electrocautery to create a stable margin for allograft integration. The F is seen at the lower left and the A at the lower right. (A, acetabulum; AL, anterolateral; F, femoral head.)

### Graft Passage

Using the MAP, a 1.8‐mm Knotless Q‐Fix anchor with suture tape (Smith & Nephew, Andover, MA) is placed on the anterior acetabular rim at the anterior junction of the segmental reconstruction (Figure 4). This first anchor is referred to as the reduction anchor. A second anchor is placed through the same portal in the rim peripheral to the native labral segment allograft junction. This anchor will serve as the first labral fixation anchor (Figure 5). The repair suture from the reduction anchor is brought through the PL portal. At this time, the graft is prepared. A ripstop suture is added to one end of the peroneus longus allograft. This is a No. 2 nonabsorbable suture that is passed in a looped and knotted fashion twice through one end of the graft. A free needle is then used to pass the repair suture from the reduction anchor through the graft and between the ripstop suture (Figure 6). The suture is then pulled to the anchor directly visualizing graft passage with the arthroscope in the AL portal (Figure 7). Once the graft is passed and reduced to the anterior junction, the reduction suture is cut. The suture from the previously placed labral fixation anchor is then passed through the native labrum and circumferentially around the anterior portion of the allograft (Figure 8).

**FIGURE 4:**
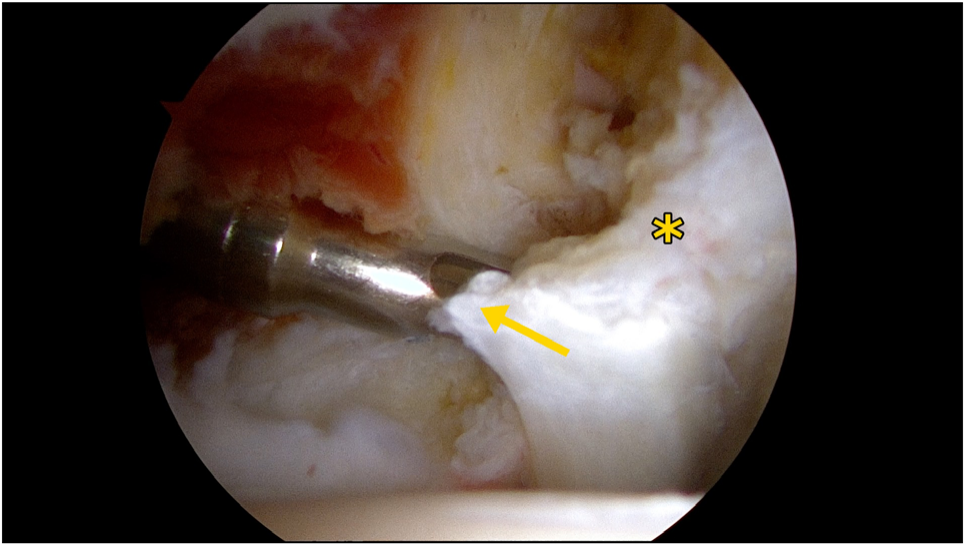
Left hip, supine position, arthroscopic view from the AL portal using a 70° arthroscope. A 1.8‐mm Knotless Q‐Fix suture anchor (Smith & Nephew, Andover, MA) is placed on the anterior acetabular rim (arrow) at the anterior junction of the segmental reconstruction, serving as the reduction anchor for the anterior end of the allograft. The adjacent native labrum is marked (asterisk). (AL, anterolateral.)

**FIGURE 5:**
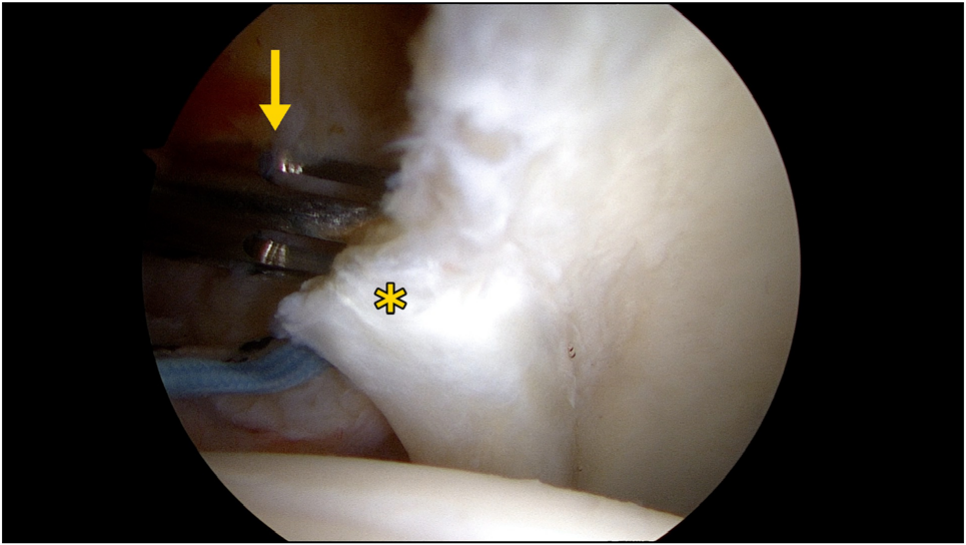
Left hip, supine position, arthroscopic view from the AL portal using a 70° arthroscope. A second anchor is placed with the drill guide (arrow) slightly proximal and posterior to the reduction anchor and peripheral to the native labral‐allograft junction. Placed before graft passage to preserve visualization, it serves as the first labral fixation anchor. The native labrum is marked (asterisk). (AL, anterolateral.)

**FIGURE 6:**
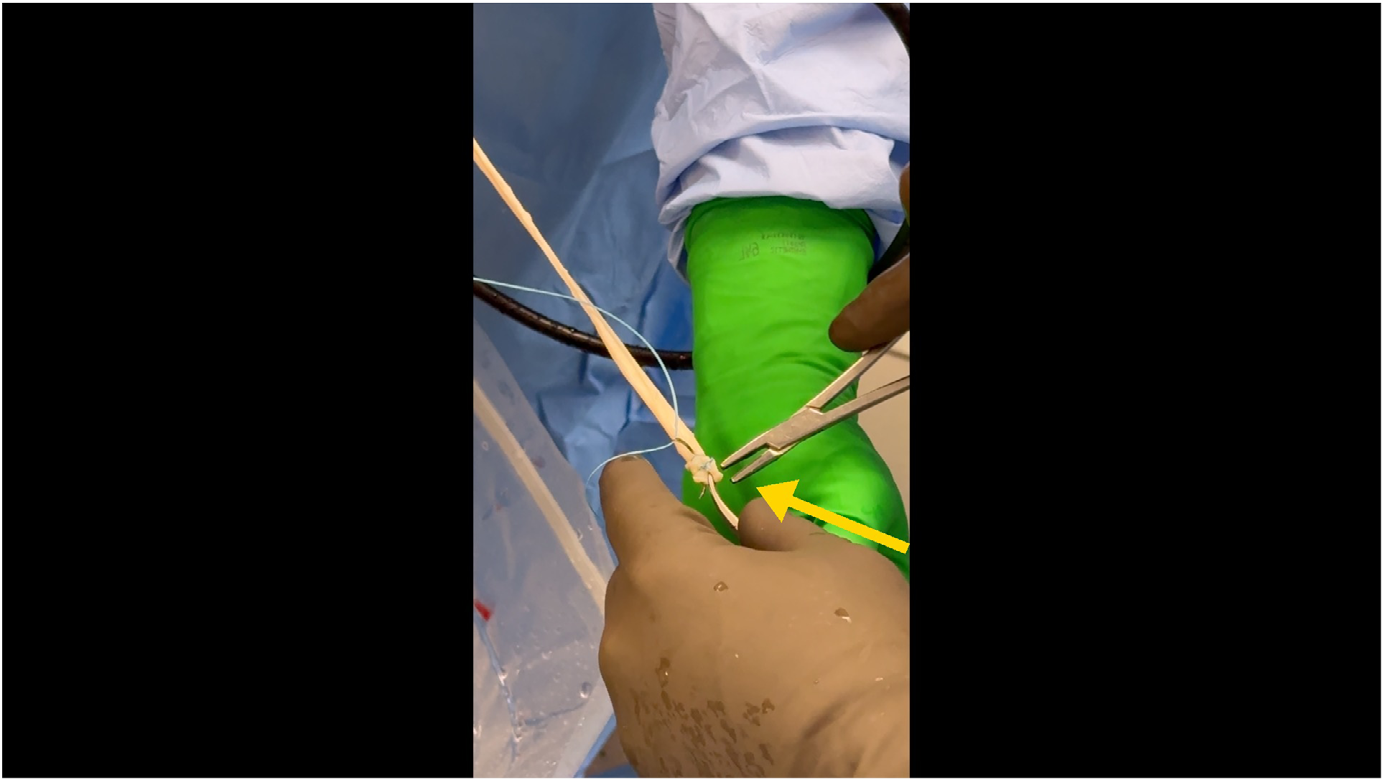
Intraoperative photograph of graft preparation for a left hip reconstruction. The peroneus longus allograft is prepared on the back table with a No. 2 nonabsorbable ripstop suture (arrow) placed in a looped, knotted configuration at the leading end. The repair suture from the reduction anchor is subsequently retrieved through the PL cannula and passed through the graft with a free needle. (PL, posterolateral.)

**FIGURE 7:**
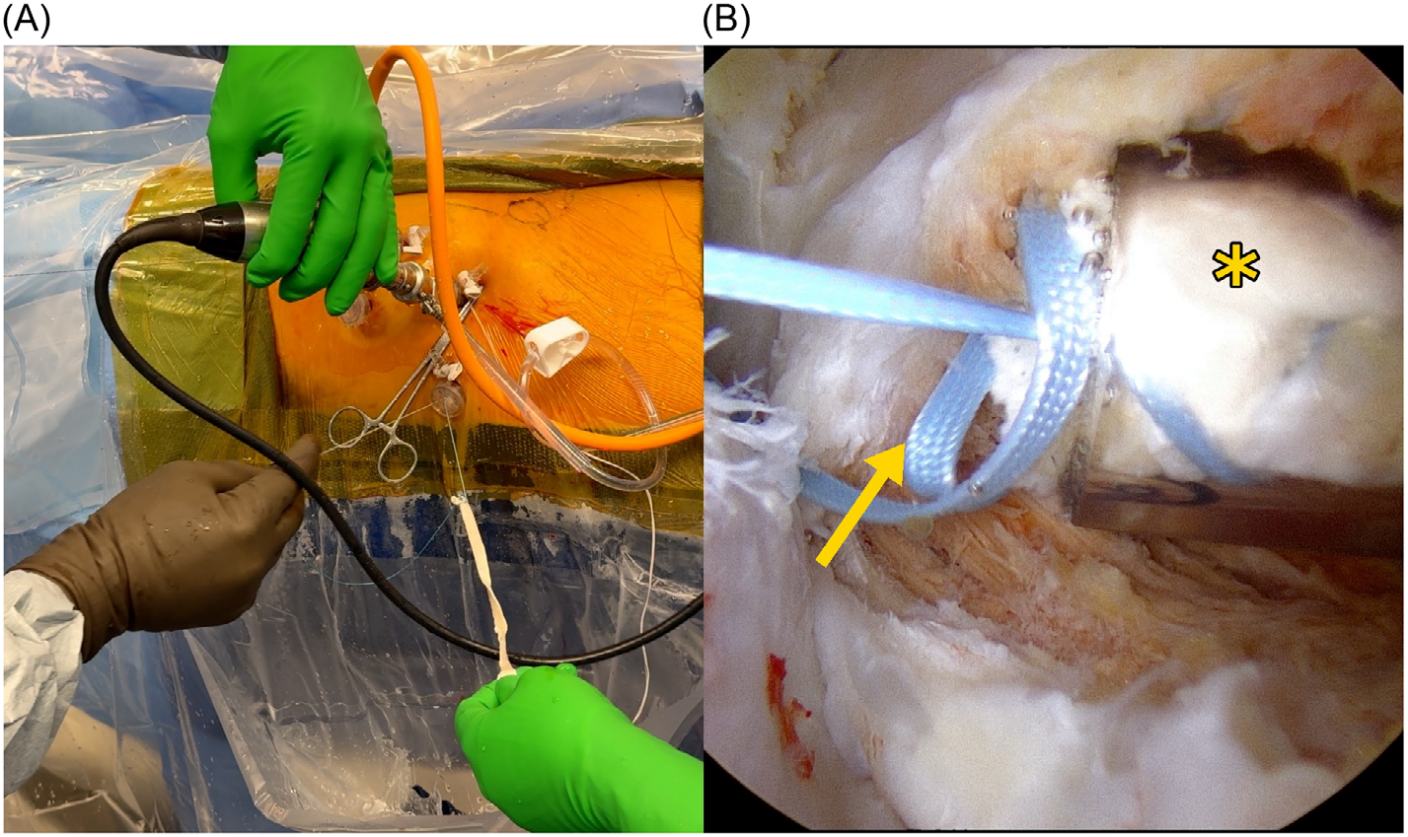
Left hip, supine position. (A) Intraoperative photograph showing the arthroscope in the AL portal; the repair suture from the reduction anchor is passed through the anchor in standard fashion, bringing the graft through the PL portal toward the anchor. (B) Arthroscopic view from the AL portal using a 70° arthroscope. The peroneus longus allograft (asterisk) is seen within the PL portal cannula, being delivered toward the anterior junction by the suture tape (arrow) from the previously placed reduction anchor. (AL, anterolateral; PL, posterolateral.)

**FIGURE 8:**
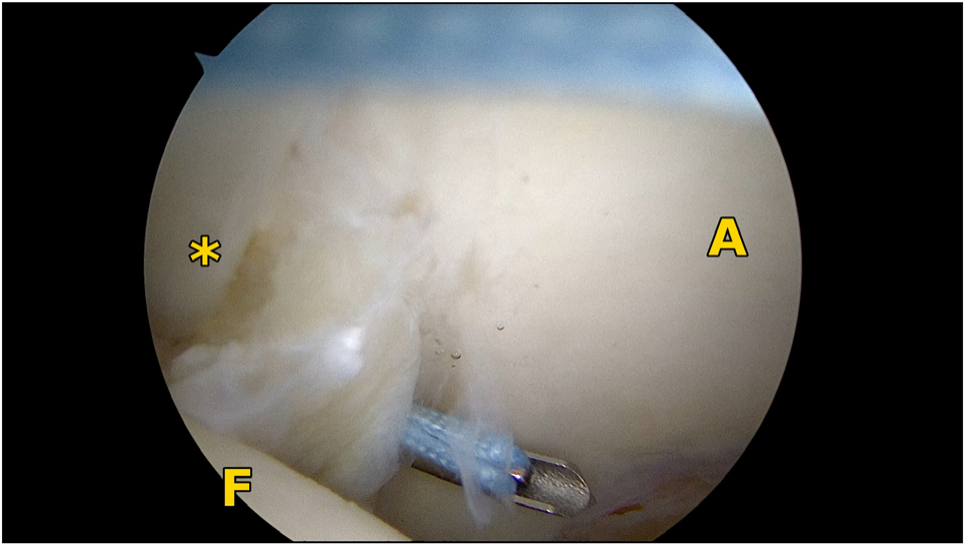
Left hip, supine position, arthroscopic view from the AL portal using a 70° arthroscope, with instrumentation through the MAP. The suture from the first labral fixation anchor is passed through the native labrum at the anterior junction and circumferentially around the most anterior portion of the allograft (asterisk), forming the first labral fixation suture. The F is seen at the lower left and the A to the right. (A, acetabulum; AL, anterolateral; F, femoral head; MAP, midanterior portal.)

### Graft Fixation

Additional anchors are placed from anterior to posterior continuing to secure the graft to the segmental defect (Figure 9A). The MAP or distal AL portals can be used for anchor placement until the most posterior portion of the reconstruction. Typical spacing for these anchors is approximately 8 to 10 mm (Figure 9B). Once the graft is reduced and adequately stabilized from anterior to posterior in the native footprint, the final posterior anchor is placed through the PL portal (Figure 10). This anchor is placed just peripheral to the junction of the graft and native labrum. The repair suture is passed through both the graft and native labrum completing the segmental reconstruction. The remaining graft is amputated using electrocautery at the posterior native labral junction and is removed from the PL portal. The segmental reconstruction is visualized from the most anterior to the most posterior extent. Traction is taken down, and the suction seal is verified (Figure 11). Femoroplasty if necessary is completed, and the capsulotomy is closed.

**FIGURE 9:**
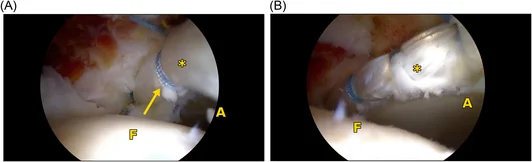
Left hip, supine position, arthroscopic view from the AL portal using a 70° arthroscope. Sequential labral fixation of the allograft. (A) The first labral fixation anchor and its associated suture tape (arrow) secure the anterior allograft (asterisk). The F is seen inferiorly and the A at the lower right. (B) Additional fixation anchors are placed sequentially from anterior to posterior, seating the graft (asterisk) along the native footprint. The F is seen at the front left and the A to the right. (A, acetabulum; AL, anterolateral; F, femoral head.)

**FIGURE 10:**
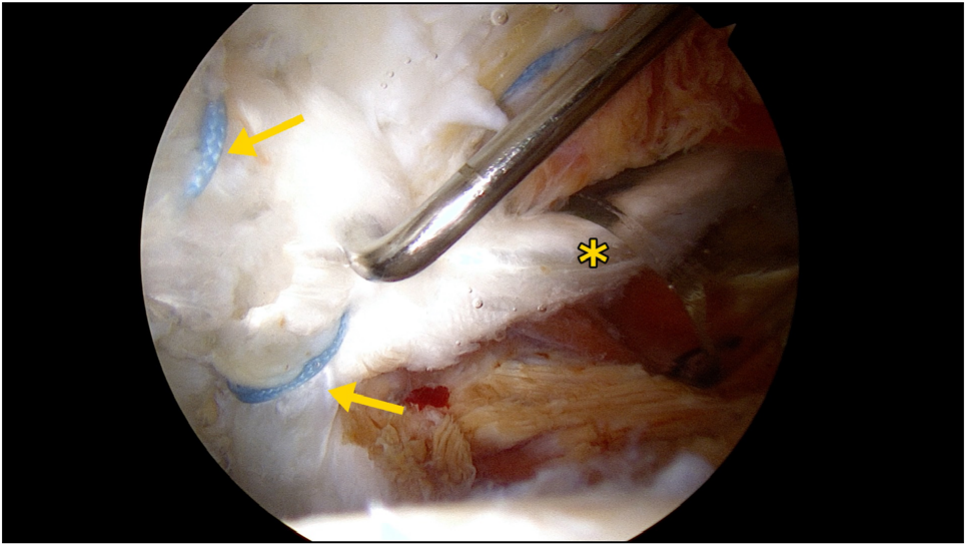
Left hip, supine position, arthroscopic view from the AL portal using a 70° arthroscope. The final anchor is placed at the most posterior junction between the allograft and native labrum, and the suture is passed through the native labrum and around the graft in the same manner as the anterior fixation. The remaining graft (asterisk) exits the PL cannula on the right of the screen and is amputated at the posterior junction; prior fixation sutures are indicated (arrows). (AL, anterolateral; PL, posterolateral.)

**FIGURE 11:**
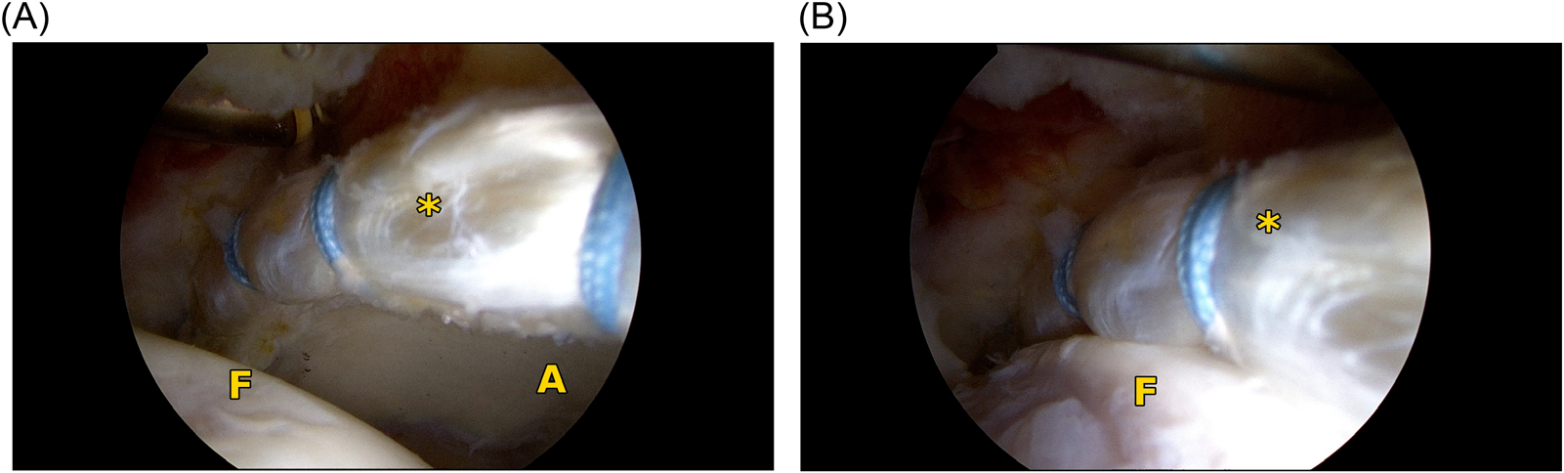
Left hip, supine position, arthroscopic view from the AL portal using a 70° arthroscope. The completed segmental reconstruction. (A) With traction maintained, the completed reconstruction (asterisk) spanning the anterior‐to‐posterior defect is evaluated. The F is seen at the lower left and the A at the lower right. (B) With traction released, the restored suction seal between the reconstructed labrum (asterisk) and the F is confirmed. (A, acetabulum; AL, anterolateral; F, femoral head.)

### Postoperative Care and Rehabilitation

Postoperatively, patients are weight‐bearing as tolerated with crutches for 2 weeks. Patients are given foam boots to be used during sleep for the first week to avoid external rotation. Passive range of motion is initiated on postoperative day 1 with a continuous passive motion machine or stationary bike with no resistance. For both exercises, hip flexion is limited to 90°. Resistance exercises are initiated immediately with isometric strengthening of the gluteal and quadriceps musculature. Isometric exercises targeting the hip abductors and adductors are initiated 2 weeks postoperatively. At that point, patients wean assistive devices and continue therapy.

## DISCUSSION

Segmental hip labral reconstruction using a peroneus longus allograft is a reproducible and effective option for patients with irreparable labral deficiency and preserved adjacent labrum. This technique provides anatomic and functional restoration of the acetabular labrum while avoiding unnecessary circumferential reconstruction. The use of a peroneus longus allograft allows for predictable sizing, consistent preparation, and avoidance of donor‐site morbidity.^7^ Additionally, segmental reconstruction lends itself to more predictable visualization for anchor and graft placement (Table 2). The success of this technique depends on appropriate patient selection, including addressing concomitant pathology and careful assessment of the chondral surface.^8^

**TABLE 2:** Pearls and Pitfalls

**Pearls**	**Pitfalls**
Careful preoperative planning of all portal locations facilitates efficient graft passage and fixation	Failure to address concomitant pathology can lead to early failure
After creation of the AL portal, all subsequent portals should be established under direct visualization	Under‐resection of native labrum can result in a poor interface between the native labrum and graft
Traction time should be minimized by preparing the graft before procedure start	Over‐resection of the native labrum can lead to difficult anchor placement and loss of healthy labral tissue
Postless traction should be used, particularly in complex or revision procedures	
Both the graft reduction anchor and the most anterior labral fixation anchor should be placed before graft introduction	

AL, anterolateral.

Hartwell et al. previously described a segmental labral reconstruction technique that uses a single working portal.^9^ However, the present technique differs in its use of a posterior portal, the posterior‐to‐anterior graft passage, and a reduction and fixation anchor placement. Overall, this current technique is suitable for larger segmental reconstructions, particularly in a revision setting, because the additional portals and passage direction allow ease of graft positioning and tension while not requiring extensive suture management with multiple prepositioned fixation anchors.

Several disadvantages of this technique should be acknowledged (Table 3). Segmental reconstruction creates 2 graft‐native labrum junctions, which represent potential biomechanical “weak spots” for future tears and graft‐native tissue mismatch.^10^ Unlike circumferential reconstruction, segmental techniques leave native labral tissue in situ, which may harbor subclinical pathology and serve as a residual pain generator. Additionally, a recent meta‐analysis by Kurapatti et al. found that circumferential reconstruction was associated with a significantly lower pooled rate of conversion to total hip arthroplasty than segmental reconstruction, although causality could not be inferred given study heterogeneity and nonrandomized designs.^11^ Regarding graft choice, the use of allograft tissue remains debated. Although recent systematic reviews have shown comparable patient‐reported outcomes between allograft and autograft,^6^, ^12^ some studies have reported higher revision rates with allograft use.^13^ A 2026 meta‐analysis of 30 studies (1968 hips) found no significant differences in most functional outcomes or revision rates between graft types, although considerable heterogeneity existed.^12^

**TABLE 3:** Advantages and Disadvantages of Segmental Arthroscopic Hip Labral Reconstruction Using Peroneus Longus Allograft

**Advantages**	**Disadvantages**
Preserves healthy native labral tissue and chondrolabral junction	Creates 2 graft‐native labrum junctions that are potential sites for future tears or tissue mismatch
Avoids donor‐site morbidity associated with autograft harvest	Does not remove all potentially damaged native labral tissue, which may serve as a residual pain generator
Consistent and predictable graft sizing with peroneus longus allograft	Allograft tissue may carry higher revision rates compared with autograft in some studies
Shorter operative time and fewer anchors compared with circumferential reconstruction	Accurate measurement of segmental defect size can be challenging
Reproducible stepwise technique with controlled graft passage via a reduction anchor	Requires advanced arthroscopic proficiency and multiportal management
Applicable in both primary and revision settings for focal irreparable defects	Not appropriate for extensive labral deficiency requiring circumferential reconstruction
Improved visualization during anchor and graft placement compared with circumferential techniques	

Despite these limitations, both segmental and circumferential labral reconstruction techniques have shown significant improvements in patient‐reported outcomes at short‐, mid‐, and long‐term follow‐up.^2^, ^5^, ^11^ This technique is particularly useful in the revision setting for focal irreparable labral lesions, especially when concomitant pathology such as capsular insufficiency and cam morphology is addressed to reduce the risk of recurrent failure.^8^

## DISCLOSURES

The author (M.B.B.) declares the following financial interests/personal relationships which may be considered as potential competing interests: M.B.B. reports royalties or licenses from Stryker; consulting fees from Smith & Nephew and Vericel; support for attending meetings and/or travel from Smith & Nephew; leadership or fiduciary role in other board, society, committee, or advocacy group, paid or unpaid, with American Orthopaedic Society for Sports Medicine (Education Committee, Fellowship Committee, Self‐Assessment Exam Committee, and council of delegates), Arthroscopy Association of North America (membership committee and fellowship committee), Orthopaedic Journal of Sports Medicine (editorial board), and American Board of Orthopaedic Surgery (Web‐Based Longitudinal Assessment Examiner). The other author (M.T.K.) declares that he has no known competing financial interests or personal relationships that could have appeared to influence the work reported in this article.
